# What’s been tried: a curated catalogue of efforts to improve access to general practice

**DOI:** 10.3399/BJGPO.2024.0184

**Published:** 2025-06-18

**Authors:** Carol Sinnott, Evleen Price, Akbar Ansari, Rebecca Fisher, Jake Beech, Hugh Alderwick, Mary Dixon-Woods

**Affiliations:** 1 The Healthcare Improvement Studies Institute, University of Cambridge, Cambridge, UK; 2 Nuffield Trust, London, UK; 3 The Health Foundation, London, UK

**Keywords:** general practice, access to primary care, systematic review, primary healthcare

## Abstract

**Background:**

Although increasing numbers of appointments are being provided, public satisfaction with access to UK general practice is declining. Previous attempts to improve access have not been systematically collated.

**Aim:**

To identify interventions to improve access to general practice in the UK, to organise these interventions into thematic categories, and to identify which aspects of access are targeted.

**Design & setting:**

Narrative systematic review.

**Method:**

A three-stage search was conducted to identify interventions used to improve access to NHS general practice. Using an iterative process, we generated thematic categories to classify interventions according to how they are intended to work. We assessed which aspects of access they addressed using the seven-feature Candidacy Framework.

**Results:**

The search identified 449 relevant sources reporting on interventions to improve access to general practice over the period 1984–2023. We generated six overarching thematic categories into which we organised these interventions: appointment innovations; direct patient access to services; increasing the number and range of professionals available in general practice; offering contacts beyond core hours, core settings, and core services; supporting patient engagement; and supporting the wider structures of general practice. We assessed which features of candidacy were addressed, with ‘permeability’ (the ease with which people can use services) emerging as the most frequent feature.

**Conclusion:**

Multiple and diverse attempts have been made to improve access in general practice over a 40-year period. This curated, thematised catalogue offers an important resource for future efforts to improve access.

## How this fits in

Access to general practice is an increasing priority for policy and practice, but previous reviews of attempts to improve access have been narrowly focused. This review comprehensively curates interventions aimed at improving access to NHS general practice over a 40-year period, organises these interventions thematically, and assesses which aspects of access are targeted using the Candidacy Framework, which characterises access as subject to multiple influences arising from people, their social contexts, and available services. The findings offer an important resource for future efforts to improve access and highlight where further attention is warranted in policy and practice.

## Introduction

General practice is the bedrock of NHS care, tasked with providing care that is comprehensive, coordinated, and continuous over the life-course for both acute and long-term conditions.^1,2^ Fulfilling these responsibilities requires that patients have reliable and ready access to general practice. However, even though more appointments are being provided than ever before,^3^ patient satisfaction with access to NHS general practice is declining.^4^ Difficulties reported by patients include problems getting through to practices, choice of available appointments, and ability to see their preferred professional.^4^ These challenges are highly consequential for patient experience and outcomes and for the satisfaction and working conditions of primary care staff.

Unsurprisingly, improving access to general practice is a policy priority.^5^ Like others before it, the new Labour government in the UK has committed to improving access to GP appointments. However, the many previous attempts that have been made to improve access have not been systematically compiled into a single resource. Previous reviews of interventions to improve access to general practice have instead been limited to specific components of access such as appointment systems,^6^ choice of services,^7^ enhancing access to best practice processes for preventive, episodic and chronic disease care,^8^ or specific groups such as rural communities.^9^ An overview of attempts to improve access to general practice that encompasses the entire patient journey, regardless of patient need or location, is likely to be of considerable value in informing future policy and practice options.

To address this need, we aimed to produce a comprehensive catalogue of interventions that have been used in efforts to improve access to general practice. With the goal of enhancing the usefulness of the catalogue, we categorised interventions thematically according to how they were intended to work. We aimed to further enhance the value of the catalogue by identifying which aspects of access were addressed by the interventions, using the Candidacy Framework^10^ to guide our assessments. Developed in 2006, the Candidacy Framework conceptualises access to health care broadly and from the perspective of vulnerable groups. Since then, the framework has been used to understand access in diverse settings including mental health,^11^ maternity care,^12^ and general practice.^13^ Candidacy describes how people identify themselves as ‘candidates’ for health care across seven features: identification of candidacy, navigation, permeability of services, appearances at health services, adjudications, offers and resistance, and operating conditions (Table 1).^10^ The framework shows how these features interact in a dynamic and contingent process subject to multiple varied influences, so avoiding a narrow conceptualisation of access (for example, focused simply on supply of appointments).^10^

**Table 1.** Summary of the seven features of the Candidacy Framework for understanding access^10^

Feature	Explanation
**Identification**	How people recognise their symptoms as needing medical attention or intervention is important to how they assert a claim to candidacy
**Navigation**	Using services requires knowledge of the available services and depends on having the practical resources to use them
**Permeability**	The ease with which people can use services depends on how many and what kinds of criteria people have to meet to use them, and on cultural and other alignments between services and individuals
**Appearances**	Appearing at services involves people making a claim to candidacy. It requires a set of competencies and socio-cultural alignments
**Adjudications**	Professional judgements about patients’ candidacy strongly influence individuals’ access to attention and interventions
**Offers and resistance**	Offers of care may be made that may be accepted (for example, utilisation) or refused (for example, non-utilisation) by individuals
**Operating conditions**	The perceived or actual availability and suitability of resources has a major impact on the local production of candidacy, as do other relevant operating conditions

## Method

### Developing a catalogue of interventions to improve access to general practice

We conducted a three-stage search to identify interventions to include in a catalogue of interventions aimed at improving access to general practice as a service (not solely GPs) in the UK that addressed any of the seven features of access in the Candidacy Framework.^10^ The focus was on the period from 1984 onwards, as this marked the introduction of mandatory vocational training for GPs and subsequent degree of standardisation of GP services.^14^

#### Search stage 1: exploratory

We started with a multi-strategy and iterative exploratory phase to scope the field and understand where information on improving access interventions might be available. First, we conducted a brainstorming exercise within the team (two academic GPs, three health service researchers, and two health policy researchers) to generate a starter list of interventions. We then undertook exploratory searching to identify interventions reported in the academic literature and other sources, including, for example, the websites of NHS England, NHS Futures, the Royal College of General Practitioners, the King’s Fund, and the Nuffield Trust. Through informal scoping, we also identified interventions in relevant peer-reviewed research articles, reviews, policy reports, programme reports, website entries, and evaluation reports for interventions in NHS general practice and scanned the citations and bibliographies of these publications.

We used the findings of these explorations to generate an initial list of interventions, providing the foundation for the more formal searching undertaken in the second stage.

#### Search stage 2: formal search and data extraction

We designed a formal systematic search in collaboration with a medical librarian using learning from the exploratory stage. The search strings related to general practice, access and actions, interventions or improvements. The search was run across three databases: MEDLINE, Embase, and the Healthcare Management Information Consortium (HMIC) (see Supplementary table S1).

After removal of duplicates, all records were reviewed independently by two researchers (CS and EP) using Covidence software to identify potentially eligible records. A full list of inclusion and exclusion criteria is available in Supplementary table S2. Briefly, we selected records for full-text review if they were written in English, from the UK, published from 1984 to the time of the review (July 2023), and provided a description of an intervention that had been used or could potentially be used to improve access to general practice, as understood using a candidacy lens. Discrepancies between reviewers on whether a paper should be selected for full-text review were resolved by consensus. We defaulted to full-text review where there was any uncertainty.

Next, two researchers (CS and EP) independently screened full texts for inclusion. Again, in case of disagreement, consensus was reached on inclusion by discussion and if necessary, a third researcher (AA) was consulted.

Data from included references were extracted by one reviewer (CS, EP, or AA), using a data extraction template (Supplementary table S3), which included fields on:

- type of source and (if research) the study design;
- the type of intervention described;
- whether the source reported on an evaluation or not;
- who or what the intervention targeted (one or more targets);
- study participants or who the intervention relates to; and
- intervention outcomes (one or more).

#### Search stage 3: policy initiatives

The third stage of the search was a sense-check, which used key central government or NHS England policy documents on primary care from 2000 onwards. The rationale for the choice of dates was the diminishing relevance of pre-2000 pre-devolution era initiatives to modern general practice, while 2000 coincides with the release of the NHS Plan,^15^ and the current era of NHS policy. Relevant policy documents were retrieved from several sources, including the Health Foundation Policy Navigator, a database of general practice policies developed as part of previous research, and from ongoing tracking of general practice policy by The Health Foundation. This searching was supplemented by a snowballing approach and by searches of the NHS England and Department of Health and Social Care websites. Relevant policy documents were searched and details on any interventions relevant to improving access to general practice were extracted by one reviewer (JB). The extracted data were validated using inclusion and exclusion criteria by another reviewer (CS). A full list of policy documents reviewed and policies extracted is available on request. Any intervention that had not been previously identified in stage one or two was added to the catalogue and used to refine categorisation.

### Categorising interventions

Starting with our explorations in stage 1, we developed and iteratively refined thematic categories for classifying interventions as the search proceeded. In two separate sorting workshops, facilitated by Miro board software, three researchers (one clinician and two health service researchers) sifted through, aligned, grouped and regrouped interventions with similar interventions in multiple cycles. The resulting categorisation was presented to and discussed with the wider research team, and then continuously refined through email correspondence. Throughout this process, the content and description of categories was amended for clarification and to account for the addition of new interventions.

### Assessing aspects of candidacy addressed by interventions

We used the Candidacy Framework to characterise which features of access (Table 1) had been addressed by the interventions we identified.^10^ Using descriptions of intervention targets and intervention outcomes in included papers, two researchers (CS and EP) independently judged which features of candidacy were addressed by the interventions. The potential direct and indirect effects of interventions were considered, showing that, for many interventions, multiple candidacy features were relevant. Through consensus discussions, we identified the feature we judged to be most impacted for each group of interventions as well as capturing all features that might be relevant.

### Patient and public involvement

This review is one element of a large integrated programme of work with a significant emphasis on patients’ views. This particular element aimed to identify, curate, and categorise a comprehensive list of interventions from the literature and did not include patient and public involvement.

## Results

**Figure 1.**
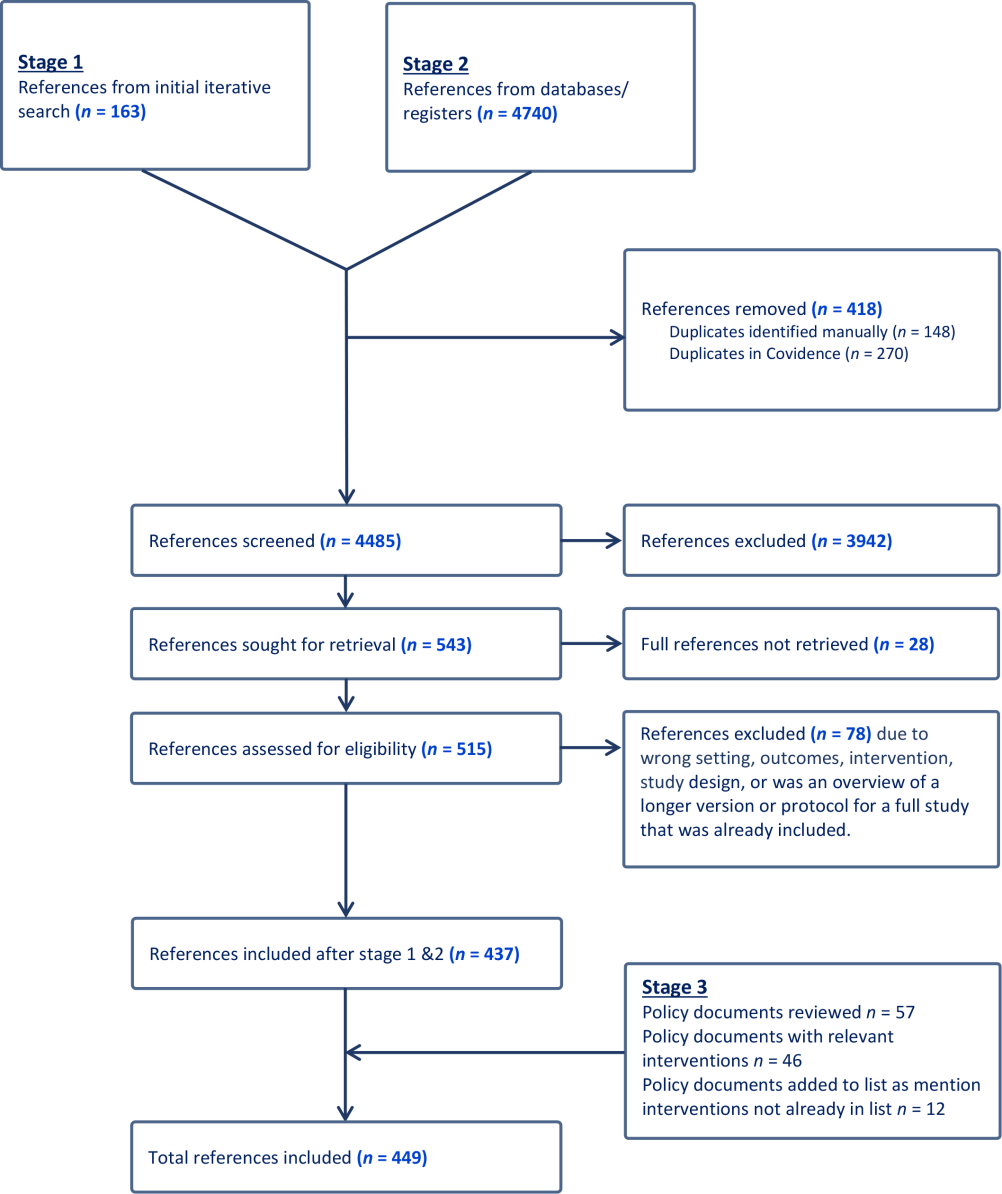
Preferred Reporting Items for Systematic reviews and Meta-Analyses (PRISMA) flow diagram of search

**Figure 2.**
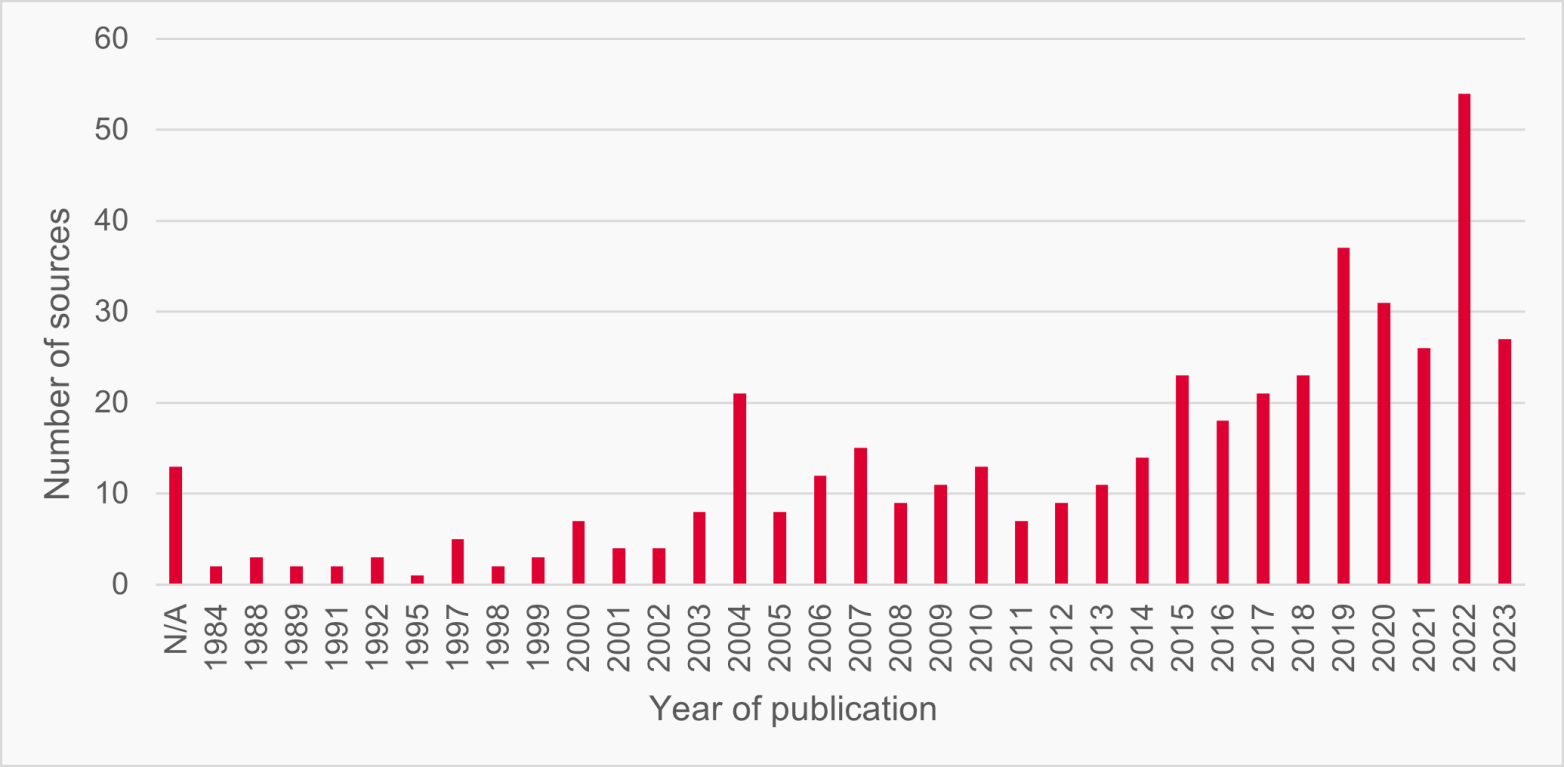
Years of publication of sources (n=449)

The Preferred Reporting Items for Systematic reviews and Meta-Analyses (PRISMA) flow diagram (Figure 1) shows the results of the three stages of the search. In total, 449 sources were included in the final catalogue. They were published between 1984 and 2023, with the majority published in the past decade (Figure 2).

**Figure 3.**
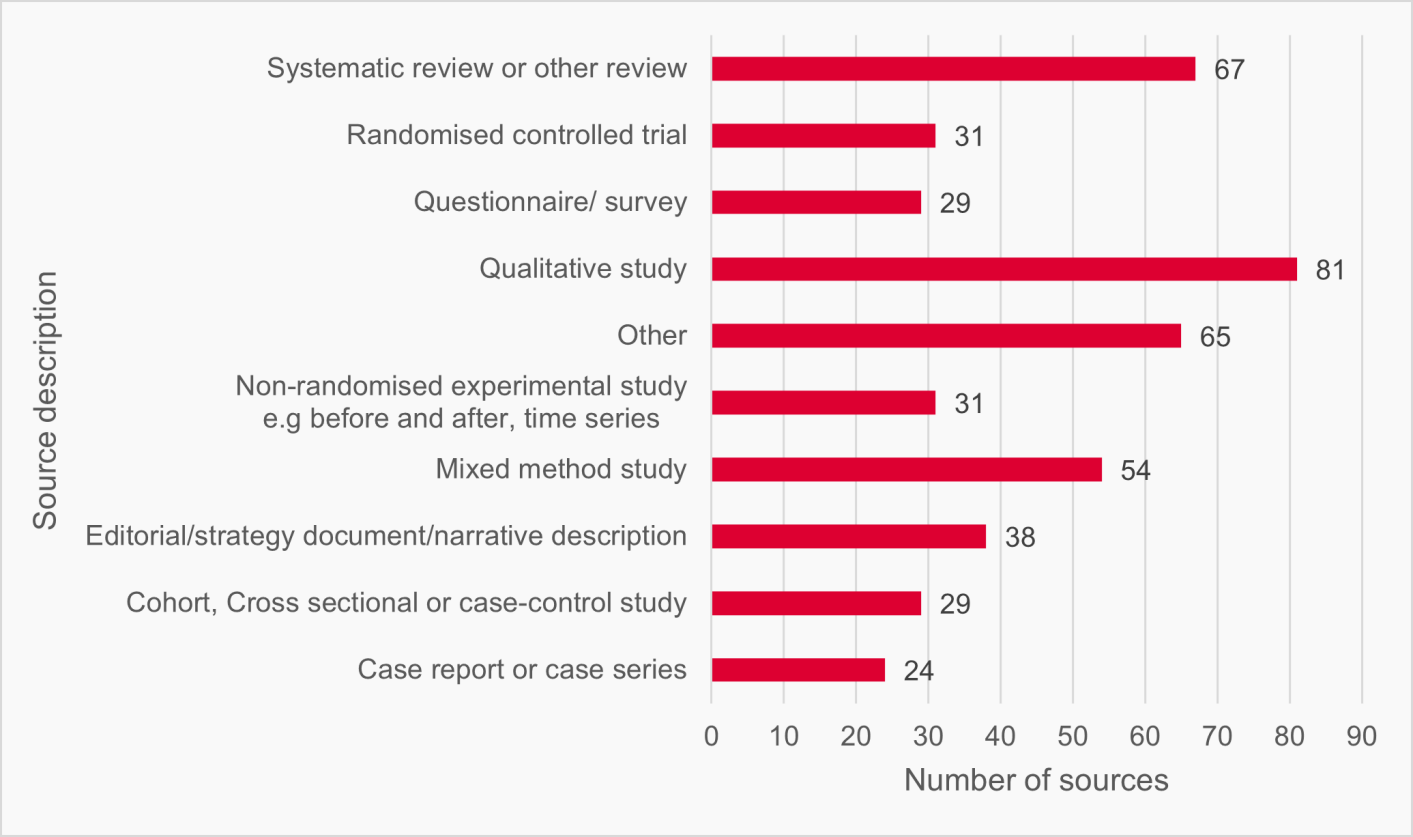
Description of sources (*n* = 449)

The records returned by our searches covered a diverse range of information sources, which can be distinguished as research and non-research sources. Research comprised academic research outputs (which we refer to as studies) and formed the majority of sources identified by our search. Non-research included government reports, strategy, or policy documents; think-tank, third-sector, medical colleges, or consultancy reports; editorials, news and opinion pieces in medical journals; guidelines, toolkits, webpages, and official statistics. Consistent with our search strategy, all included sources, research and non-research, described one or more interventions relevant to improving access to general practice. Where sources were studies, they most frequently referred to qualitative (*n* = 81), systematic or other reviews (*n* = 67), or mixed-methods studies (*n* = 54) (Figure 3).

Overall, approximately 103 different types of interventions were described across the 449 sources. We generated six overarching thematic categories into which these interventions were organised, as follows:

- Appointment innovations;
- Direct patient access to services that remove need to access general practice;
- Increasing the number and range of professionals available to see patients within general practice;
- Offering contacts beyond core hours, core settings, and core services;
- Supporting patient engagement, empowerment, and education;
- Supporting the internal and wider structures of general practice.

We also generated a further 22 sub-categories (Table 2 and Supplementary table S4). Detailed descriptions of how interventions in each subcategory might be expected (or were intended) to improve access to general practice, along with examples of interventions, are provided in Supplementary table S4. Most frequently, interventions related to innovations in appointment systems (*n* = 60 sources), broadening the range of professionals in general practice (*n* = 49 sources), expansion of out-of-hours care external to practices (*n* = 46 sources), and providing patients with contacts that were not appointments (*n* = 45 sources) (Supplementary table S4).

**Table 2.** Summary of categorisation and the features of the Candidacy Framework addressed by each subcategory

Main categories	Subcategories	I	N	P	Ap	Ad	Of	OC
Appointment innovations	Triage		2	1				
	Telehealth			1	2			
	Restructuring appointment systems			1	2			
	Offering patient contacts that are not appointments	2	2	1	2			
Direct patient access to services that remove need to access general practice	Community pharmacists			1				2
	Self-referral pathways			1				
Increasing the number and range of professionals available to see patients within general practice	Expansion and diversification of skill-mix		2	1		2		2
	Improving GP recruitment and retention			2				
Offering contacts beyond core hours, core settings, and core services	Extended hours services provided by GPs within their own practice or practice network			1				
	GP services external to a patient’s practice or local practice network			1				
	Enhanced services within practices	2		1				
	Expanded or re-organised services within the wider community	2	2	1	2			
Supporting patient engagement, empowerment, and education	Educational initiatives targeting patients	1	2	2	2			
	Digital resources for patients	1	2					
	Practice-level interventions or interventions targeting practice staff and professional behaviour	2	2	2	1	2	2	
	System-level interventions targeted at patients		1	2	2			2
Supporting the internal and wider structures of general practice	Making existing processes in practices more efficient							1
	Reducing the burden of bureaucracy in general practices							1
	Interventions to ensure general practices at high risk of closing stay open							1
	Financial mechanisms for improving access							1
	Contracting and commissioning to shape provider markets							1
	Changes to the scale or model of general practice (not otherwise included)							1

Ad = adjudications. Ap = appearances. I = identification of candidacy. N = navigation. P = permeability. OC = operating conditions. Of = offers and resistance. See description of each feature in Table 1. ‘1’ indicates the candidacy feature most addressed by an intervention, ‘2’ indicates secondarily affected features.

**Figure 4.**
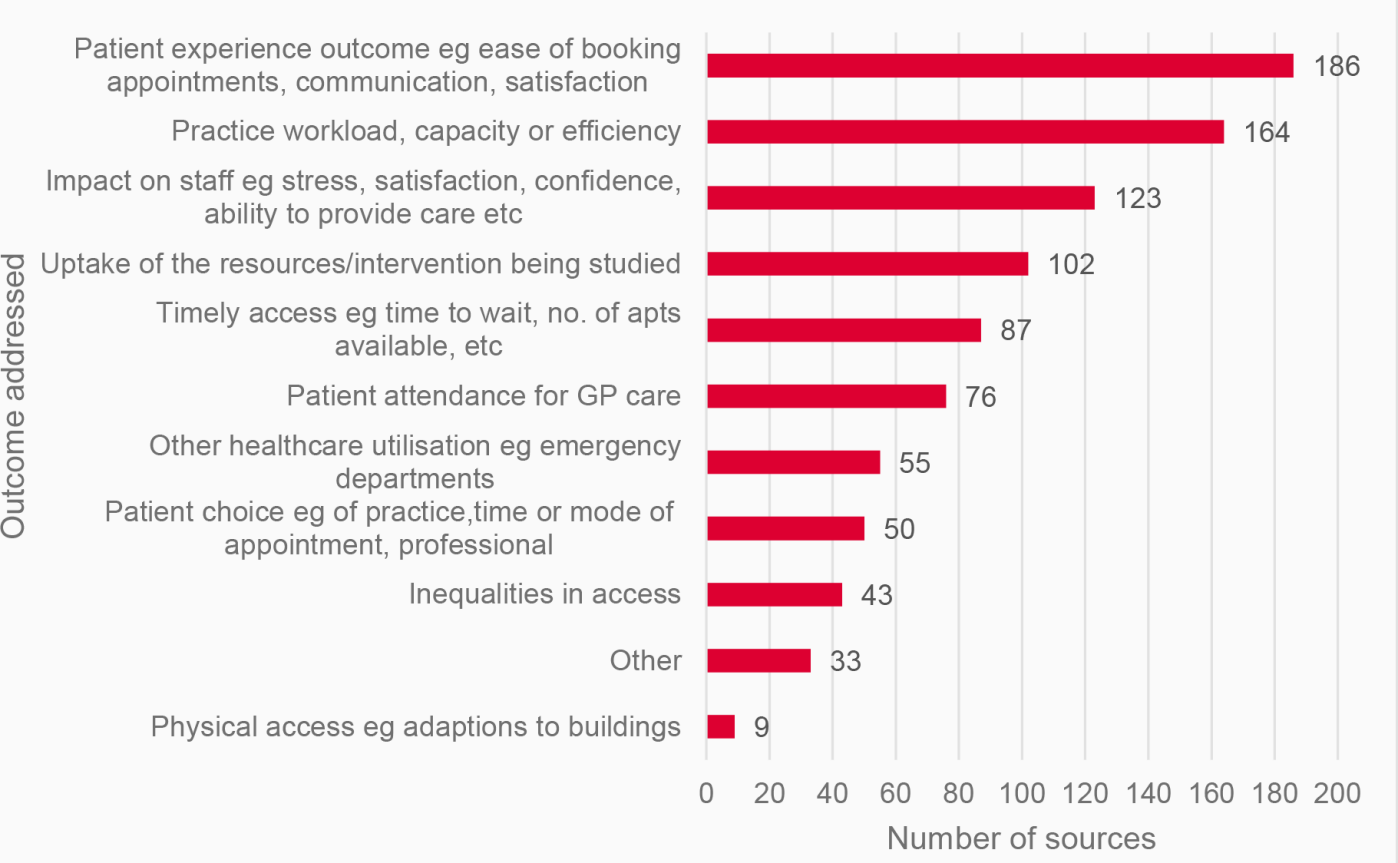
Outcomes addressed by included sources. ^a^Sources could address multiple different outcomes. Other outcomes related to: self-management (*n* = 7), continuity (*n* = 6), costs (*n* = 6), medication-related (*n* = 4); implementation outcomes (*n* = 1); access for underserved or seldom heard groups (*n* = 2), other patient and staff impacts (*n* = 4), safety (*n* = 2), new guidance (*n* = 1).

The outcomes addressed by interventions most frequently related to patient experience outcomes such as ease of booking appointments, communication, satisfaction (*n* = 186), practice workload, capacity, or efficiency (*n* = 164) or impact on staff such as stress, satisfaction, confidence, or ability to provide care (*n* = 123) (Figure 4).

Of the seven features of candidacy, ‘permeability’ of services was most frequently addressed by the interventions in the catalogue (Table 2). Permeability refers to the ease with which people can use services. More permeable services do not require extensive personal resources to be mobilised to use the service and do not demand ‘qualifications of candidacy’ (for example, referrals). Interventions that impacted on permeability included triage, telehealth, more appointments through increased skill-mix such as the introduction of physician associates or increased use of community pharmacy, walk-in centres, and urgent care clinics. The candidacy feature least frequently addressed was ‘offers and resistance’, which describes individuals’ decisions to accept or refuse offers of care (for example, prescriptions, referrals, advice). Interventions addressing this feature were aimed at supporting healthcare professionals to better engage patients in consultations and shared decision making.

We found that some interventions had potential to affect multiple features of the Candidacy Framework and may do so in ways that mean some aspects of access are improved while others deteriorate, or that have variable consequences for different groups. For example, digital triage can potentially improve permeability for some, making it easier to access care, but potentially reduce permeability for others by demanding more personal resources such as digital literacy and reliable access to the internet.

Other unintended negative consequences of interventions to improve access include incentivisation schemes for patients with certain index conditions (for example, Quality and Outcomes Framework; QOF), which can potentially constrain access for those without these conditions. Interventions such as educational initiatives targeting patients are intended to support patients’ navigation of health services, but might create the perception of a care threshold to merit attending a general practice and deter individuals with vague or ill-defined symptoms from attending (so affecting *’identification of candidacy*’).

## Discussion

### Summary

Our review has produced a comprehensive catalogue of the many attempts to improve access to general practice in the UK over more than 40 years. Guided by a framework for understanding access to health care that considers the entire patient journey and the influences on it, the thematic classification of these attempts adds to the value of the catalogue for policymakers, researchers, clinicians, and others.

### Strengths and limitations

The strengths of this review include its extensive, multi-stage search strategy, the involvement of a multidisciplinary team in its design and conduct, and an iteratively developed classificatory scheme to systematically curate a large and sprawling set of sources on improving access. We included all 400+ relevant references identified in our search in the final to provide researchers and policymakers with an extensive, comprehensive resource to inform and guide future research. We limited our search to sources from a UK context, and, while we sought to be comprehensive, we did not seek to be exhaustive. The searching is therefore unlikely to have picked up every attempt to improve access (including more local efforts, for example).

At the same time, many of the initial citations returned by our comprehensive search were only tangentially related to the search terms used. From this large initial number of citations, we selected papers that addressed the broad understanding of access offered by the Candidacy Framework. Some of these papers might not have been selected had we used a more conventional approach to understanding of access (for example, based solely on supply of appointments). Adopting this broader conceptualisation of access led to many questions and challenges within the research team on what studies were in fact relevant or not, but is likely to have led to a richer and more holistic understanding of issues relevant to general practice access that may be helpful to others.

Important policies from across the UK devolved nations were identified by the stage 2 search (for instance the Scottish *Shaping the Future Together: Remote and Rural General Practice Working Group report*; the *Making it Easy — A Health Literacy Action Plan for Scotland*;^16^ and the Welsh Choose Pharmacy programme). Our policy review was initially intended as a sense-check for this stage 2 search. Based on the expertise and capacity within the team, and the fact that England has the most extensive policy documentation, we focused on English policies. Only a small minority of the English policies we identified through this process ultimately contributed new interventions to the list. Accordingly, although general practice policy is not identical across the four UK nations, we believe that a full review of policies for each devolved nation would not have had a major effect on conceptual refinement of the list of interventions. Differences in policies related to access to general practice across the devolved nations is a question that could be addressed by future research.

None of the interventions that we reviewed were designed, implemented, or evaluated with candidacy in mind, so our assessment of the candidacy features addressed by included sources were based on judgements made within the research team. In practice, interventions might have spill-over or other effects that would not have been anticipated during our process of aligning interventions to features of the Candidacy Framework. To simplify presentation, the seven features of candidacy have been presented discretely, but in practice these features often overlap. Further, the boundaries have become blurred because of recent changes, including, for example, the digitalisation of many healthcare interactions in general practice and broadening of skill-mix.

We did not set out to conduct a critical appraisal of study findings, nor aim to assess the effectiveness, advantages and disadvantages of particular interventions, but this review lays the foundation for future work of this nature.

### Comparison with existing literature

Prior reviews of attempts to improve access to general practice have focused on specific dimensions or narrower understandings of access. Eccles *et al* ^6^ explored different access systems, defining them as providing access to an appointment for a consultation. This pragmatic but narrow definition excludes many of the influences on access characterised by the Candidacy Framework and is perhaps indicative of a practice-focused rather than a patient-oriented perspective. It also focused on academic literature, thus excluding policy papers and NHS England websites.

Tan and Mays reviewed research that evaluated 10 government initiatives to expand access to primary care in the UK.^7^ The 19 included studies of these 10 initiatives addressed extended and out-of-hours access to primary care, urgent care, and walk-in centres, advanced access appointment systems, enhanced availability of telephone and online health information and the GP Choice pilot, between the years 1997 and 2013. Demonstrating the value of looking to the past to learn for the future, the authors found that new services appeared to increase overall demand rather than inducing substitution between services, suggesting that enhancing access to existing provision may have greater potential to improve access than developing new services.

Other reviews have concentrated on the implications of interventions for different healthcare systems such as Canada^17^ or Australia^8^ or were conducted too long ago to reflect some of the recent developments in NHS general practice.^18^

### Implications for research and practice

Future attempts to improve access to general practice at both practice and policy level can benefit from our curated catalogue of what has already been tried. Our work, like others, also suggests that a broader conceptualisation of access is much needed.^19^ By drawing on the Candidacy Framework,^10^ the diverse influences on access became clearer. Our finding that most interventions target the candidacy feature of permeability — the ease with which people can use healthcare services — rather than addressing the precursors, sequelae, and wider influences on access to health care suggests that interventions to improve access have been operationalised in overly limiting ways.^10^ For practices and practice networks, the catalogue illuminates the broad range of targets and strategies that are available across the patient journey for enhancing patients’ experiences of access. Adopting a more holistic view of access can help policymakers move beyond ‘supply’-focused solutions to consider where other improvements to access are needed, to understand why previously attempts have not had sufficient impact and to identify alternative solutions, including those that promote equity.

Given the recursive nature of patients’ relationships with primary care, shifting the policy emphasis from the current preoccupation with permeability would enable better recognition of factors that create failure demand (and associated unnecessary return visits) by focusing attention on how patients identify themselves as candidates, how decisions are made and communicated during patient–professional interactions, how patients’ questions and concerns are addressed, and how more satisfying experiences of care could be delivered.^20^ These kinds of influences on access were only rarely considered in the interventions in our catalogue. There was also a dearth of interventions addressing the features of ‘identification of candidacy’ and ‘navigation’, despite their relevance in recent literature on patient experience of access^21–24^ and healthcare policy reviews on access.^25^

A potential benefit of using the Candidacy Framework to design or evaluate interventions to improve access to general practice — at policy or practice level — is its ability to facilitate assessment of the broader implications of interventions, including both positive and negative effects. For example, while telehealth is intended to increase capacity in general practice and thus improve permeability, it might have negative implications for patients’ appearances and for health professional adjudications that impact on perceptions of access.^26^ Expanded offerings for out-of-hours general practice care may be envisioned to improve permeability, but may cause problems of navigation for some cohorts of patients.^27^ Translators may improve patient experiences of appearances and adjudications, but the longer appointments needed may negatively impact on the permeability experienced by others.^28^ Also evident is that an indicator set^29^ for research on access to general practice would facilitate better comparisons of interventions across settings.

In conclusion, we were successful in compiling a catalogue of interventions that have been used in efforts to improve access to general practice in the NHS over the period 1984–2023,^30^ in organising them into thematic categories, and in classifying the features of candidacy targeted by each intervention. A wide variety of interventions have been used to address access problems in general practice, with many tackling the permeability of general practice and fewer considering relational dimensions of care. These findings have considerable value in informing future efforts, especially those of the UK’s new government, to improve access and patient experiences of access to general practice.

## Data

The dataset relied on in this article is available from the corresponding author on reasonable request.
